# Subscapularis Partial Thickness Tears by Yoo and Rhee Classification: Identifying MRI Predictors for Type IIB, Requiring Surgical Repair

**DOI:** 10.3390/diagnostics15212670

**Published:** 2025-10-22

**Authors:** Yoonsang Lee, Seul Ki Lee, Jee-Young Kim

**Affiliations:** Department of Radiology, St. Vincent’s Hospital, College of Medicine, The Catholic University of Korea, Seoul 06591, Republic of Korea

**Keywords:** subscapularis, rotator cuff tears, shoulder, magnetic resonance imaging, classification

## Abstract

**Objectives**: This study aimed to assess the significance of a novel subclassification for partial thickness tears of the subscapularis tendon (SSC) by Yoo and Rhee and to identify key MRI findings predictive of the newly adopted surgical indicator (Yoo type IIB). **Methods**: Between June 2021 and January 2024, 190 patients undergoing preoperative MRI and arthroscopic rotator cuff repair were enrolled. Patients with arthroscopically confirmed Lafosse type 1 tears (n = 148) who underwent debridement were included. Preoperative MRIs were retrospectively evaluated for SSC tear according to Yoo and Rhee classification, muscle atrophy, fatty infiltration, lesser tuberosity cyst, and long head of the biceps (LHBT) pathologies. Patients were divided into the control (Yoo type I + IIA) and study (Yoo type IIB) groups, and significant associations of MRI findings between the groups were investigated. **Results**: Among Lafosse type 1 patients, the control group (Yoo type I [n = 70] and Yoo type IIA [n = 41]; n = 111; mean age, 61.8 years ± 9.6, 48 men), and the study group (Yoo type IIB, n = 32; mean age, 66.2 years ± 7.8, 16 men) showed significant differences in age (*p* = 0.017), but not in gender (*p* = 0.634). Preoperative MRI findings, including muscle atrophy (*p* < 0.001), fatty infiltration (*p* < 0.001), lesser tuberosity cyst (*p* = 0.033), and LHBT pathologies (full thickness tear, *p* = 0.040; partial thickness tear, *p* < 0.001; tendinosis, *p* = 0.003; subluxation, *p* < 0.001), differed significantly between the groups. Multivariate analysis identified muscle atrophy (odds ratio [OR] = 33.83, *p* = 0.008) and LHBT subluxation (OR = 22.83, *p* < 0.001) as independent predictors for Yoo type IIB. **Conclusions**: In partial thickness tears of SSC, significant MRI differences were found between the Yoo and Rhee classifications. Notably, muscle atrophy and LHBT subluxation were valuable indicators for predicting Yoo type IIB as a surgical indication.

## 1. Introduction

The subscapularis tendon (SSC) plays a pivotal role in internal rotation and glenohumeral stability as a crucial component of the rotator cuff complex [1]. Despite its importance, SSC tears are frequently underdiagnosed, particularly when limited to partial thickness lesions [2,3]. These subtle tears are often difficult to detect on conventional magnetic resonance imaging (MRI) and may be underestimated during arthroscopic evaluation [4]. Consequently, the diagnosis and management of partial thickness SSC tears remain a clinical challenge, with uncertainty regarding which lesions warrant surgical repair versus conservative treatment [5].

Multiple classification systems have emerged to characterize SSC tears. The Lafosse classification has long been the standard for describing SSC tendon pathology in arthroscopy, ranging from type 1 (partial lesions of the superior third of the tendon) to type 5 (complete rupture with humeral head subluxation or severe fatty degeneration) [5]. However, Lafosse type 1 lesions—partial tears—are particularly heterogeneous, and the absence of consistent imaging-based subclassification creates a clinical gap in determining which patients may benefit from surgical intervention versus conservative management [6,7].

To address this gap, Yoo and Rhee proposed a novel classification based on the facet anatomy of the SSC footprint, refining Lafosse type 1 lesions into Yoo and Rhee type I, IIA, and IIB [8]–(Yoo type I) leading-edge fraying or longitudinal split; (Yoo type IIA) ≤ 50% detachment of the first facet; (Yoo type IIB) > 50% detachment of the first facet without complete lateral band disruption. Higher-grade tears (Yoo types III–V) reflect progressively more extensive tears, including full thickness and retracted lesions. Notably, Yoo type IIB lesions have emerged as a potential surgical indicator, representing a critical threshold where structural compromise of the tendon may necessitate repair despite the absence of a full thickness tear [8]. Importantly, subsequent studies have demonstrated the clinical and imaging utility of this system. A multicenter MRI study reported that conventional MRI achieved high diagnostic performance for differentiating Yoo type IIA from Yoo type IIB or higher-grade tears, with good interobserver agreement [9]. Recent reviews have further emphasized that the Yoo type IIB lesion represents a clinically significant threshold, often associated with surgical indication [6]. Clinical studies also support the distinction, showing that patients with Yoo type IIB tears demonstrate greater functional impairment compared with those with Yoo type IIA [10]. Collectively, this facet-based approach provides a more clinically meaningful framework for improving MRI-based detection of clinically relevant partial thickness SSC tears and supporting surgical treatment decisions.

Despite its promise, reliable preoperative MRI predictors for Yoo type IIB lesions remain undefined. Accordingly, the purpose of this study was to assess the significance of the novel subclassification for partial thickness tears of the SSC by Yoo and Rhee, with a focus on identifying key MRI findings predictive of the newly adopted surgical indicator, Yoo type IIB. We hypothesize that MRI signs such as muscle atrophy and long head of biceps tendon (LHBT) instability may serve as independent predictors of Yoo type IIB, thereby enhancing preoperative diagnostic accuracy and assisting clinicians in operative planning.

## 2. Materials and Methods

This retrospective study was approved by the institutional review board of our institution, and the requirement for informed consent was waived due to the retrospective nature of the study.

### 2.1. Patient Population

**Figure 1:**
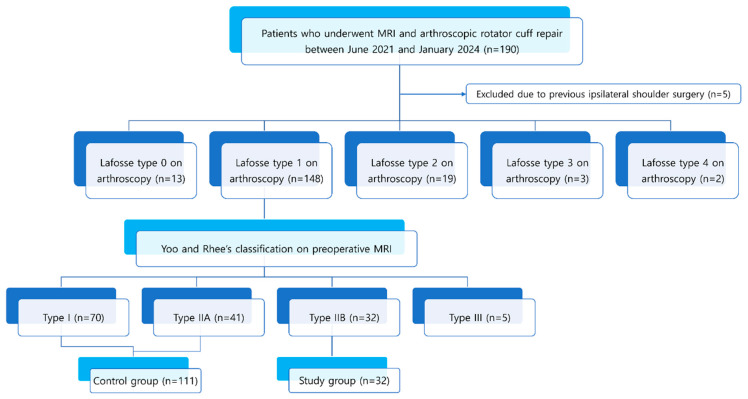
Flowchart of study population and patient recruitment process.

This retrospective study was conducted between June 2021 and January 2024, involving patients who underwent preoperative MRI followed by arthroscopic rotator cuff repair. A total of 190 patients were initially enrolled in the study. Five cases were excluded because of previous shoulder surgery on the affected side. The arthroscopic assessment served as the gold standard for confirming SSC tears. Following arthroscopic confirmation, 148 patients with Lafosse type 1 SSC tears who underwent debridement were included in the final analysis. Patients were then stratified into two groups based on the Yoo and Rhee classification, comprising 111 cases in the control group (Yoo type I and type IIA) and 32 cases in the study group (Yoo type IIB). We focused on Lafosse type 1 lesions, as partial thickness tears are the most clinically ambiguous and diagnostically challenging. The Yoo and Rhee system enabled subclassification of Lafosse type 1 lesions into types I/IIA and type IIB. Our primary aim was to identify MRI predictors of Yoo type IIB, which has recently been recognized as a potential surgical indicator. Therefore, lesions beyond this spectrum were intentionally excluded to maintain focus on this clinically relevant research purpose. Demographic data, including sex and age, were obtained from the medical records. A flowchart of the patient selection process is shown in Figure 1.

### 2.2. MRI Protocol

MRI examinations were performed with 3.0-tesla scanners (Magnetom Verio, Siemens Healthineers, Erlangen, Germany, or Ingenia, Philips Healthcare, Best, The Netherlands) with a dedicated shoulder coil. The imaging protocols included spin-echo T1-weighted (TR/TE range, 623/11) and spin-echo T2-weighted with and without fat suppression (TR range/TE range: 4000–6200/63–76). The images were obtained in three orthogonal planes: axial, oblique coronal, and oblique sagittal. Axial images extended from the superior margin of the acromion to the inferior aspect of the glenoid fossa. Oblique coronal images were acquired parallel to the long axis of the supraspinatus tendon, as identified on the axial plane. Oblique sagittal images were obtained parallel to the glenoid surface and oriented perpendicular to the oblique coronal plane. These protocols were standardized across all vendors, following ESSR (European Society of Musculoskeletal Radiology) guidelines for shoulder imaging.

### 2.3. MRI Analysis

All preoperative MRI studies were retrospectively evaluated by two radiologists with 2 and 13 years of experience (Y.L. and S.K.L.) who were blinded to the patient’s clinical information and arthroscopic findings. In cases of disagreement between the two independent radiologists, a consensus meeting was held to reach the final interpretation. If consensus could not be achieved, the senior radiologist (J.-Y.K.)’s assessment was considered definitive.

**Figure 2:**
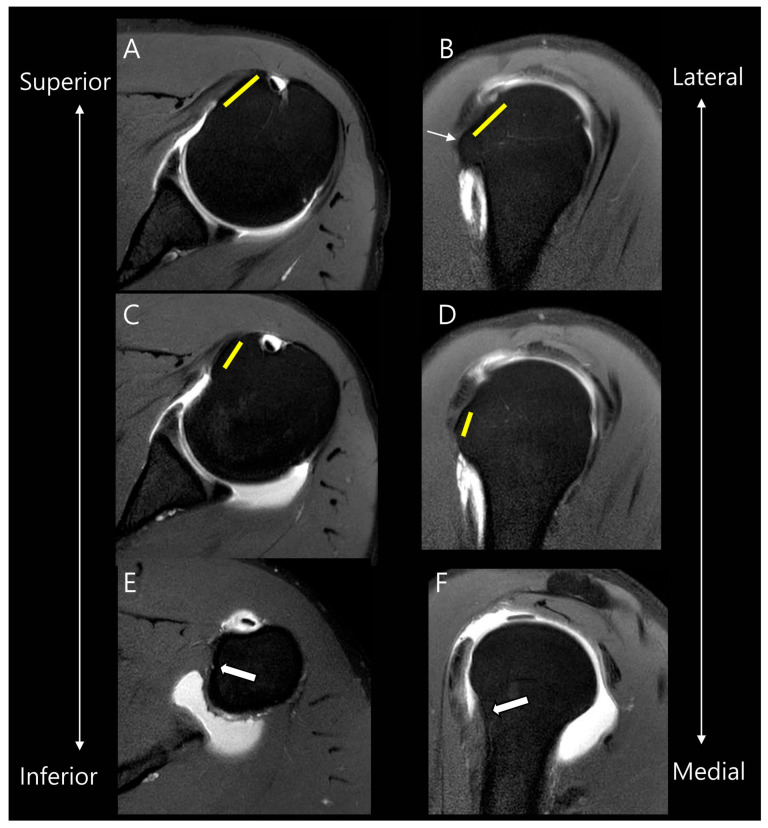
Anatomy of facets of the subscapularis tendon on MR images. On oblique axial and oblique sagittal MR images, the first facet (yellow lines in (**A**,**B**)) is located more superiorly and laterally compared to the second facet (yellow lines in (**C**,**D**)). They are separated by a bony ridge (thin arrow in (**B**)). The following third and fourth facets represent the large muscular insertion in the inferior attachment (thick arrows in (**E**,**F**)).

Before evaluating the SSC tear, we reviewed the anatomy according to the four facet theory of the SSC footprint as described in the Yoo and Rhee classification [8]. In this theory, the first two facets represent 60% of the total footprint, corresponding to the tendinous portion of SSC attachment. The first facet corresponds to the upper one-third of the SSC footprint as described by Lafosse [8]. On axial images, it appears relatively wide and flat, while on sagittal images, it demonstrates a horizontal orientation [9]. Anatomically, the first facet is positioned most superiorly and laterally. The second facet corresponds to the upper two-thirds of the SSC footprint according to Lafosse. On axial images, it appears relatively narrow, and on sagittal images, it is oriented more vertically [9]. It is separated from the first facet by a bony ridge and is located more inferiorly and medially relative to the first facet. In comparison, the following third and fourth facets represent the large muscular insertion of the SSC, which aligns with the caudal one-third described in the Lafosse classification [8]. Figure 2 presents an illustration correlating the facet theory with actual MR images.

**Figure 3:**
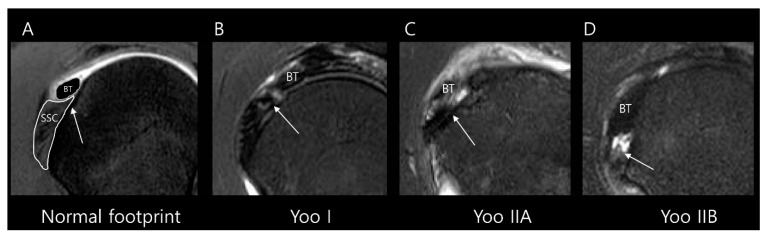
Subclassification of Lafosse type 1 subscapularis tendon (SSC) partial thickness tears according to the Yoo and Rhee classification. On T2-weighted oblique sagittal images with fat suppression, (**A**) a normal SSC footprint shows a thin tendinous slip (arrow) in the superior portion, which is closely related to the stability of the biceps tendon (BT). Note that most partial thickness tears (Lafosse type 1) originate from this tendinous slip. (**B**) Yoo type I tear shows fraying or a longitudinal split at the leading edge (arrow). (**C**) Yoo type IIA tear is defined as the detachment of less than 50% of the first facet (arrow). (**D**) Yoo type IIB tear is defined as the detachment of more than 50% of the first facet (arrow).

We collected SSC partial thickness tears belonging to Lafosse type 1 on arthroscopy and subclassified them according to the Yoo and Rhee classification (Table 1) using the following parameters (Figure 3) [11]: Yoo type I, leading-edge fraying or longitudinal split; Yoo type IIA, ≤50% detachment of the first facet; Yoo type IIB, >50% detachment of the first facet. It should be noted that, in the Lafosse classification, the surgical indication is defined as a type 2 tear, corresponding to a full thickness tear of the upper one-third. In contrast, the Yoo and Rhee classification defines the surgical indication as Yoo type IIB.

**Figure 4:**
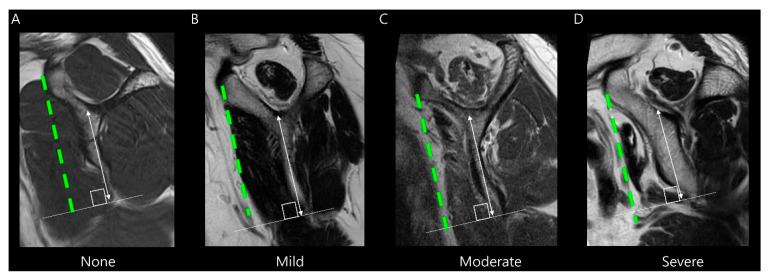
Evaluation of subscapularis muscle atrophy on T2-weighted oblique sagittal images. The grade is determined by the amount of muscle above or below a virtual line (green dashed line) drawn from the superior border of the coracoid process to the inferior margin of the subscapularis muscle at the level of the scapular inferior tip (solid line), parallel to the long axis of the scapular body (double-headed arrow). (**A**) None, (**B**) Mild atrophy, (**C**) Moderate atrophy, and (**D**) Severe atrophy.

Subscapularis muscle atrophy was evaluated using the Warner classification [12] and the method described by Seppel et al. [13]. The muscle area of the subscapularis was measured in the “Y-position” on the oblique sagittal plane, defined as the most lateral image where the scapular spine contacts the body of the scapula. A virtual reference line was drawn from the superior border of the coracoid process to the inferior margin of the subscapularis muscle at the level of the scapular inferior tip, parallel to the long axis of the scapular body (Figure 4). If the muscle is convex above the line, there is no atrophy. If the muscle contour is even with the line, mild atrophy exists. If the contour of the muscle is concave below the line, moderate atrophy is present. If there is barely any muscle visible, severe atrophy exists [12]. For statistical analysis, we categorized muscle atrophy into two categories: non-mild and moderate–severe degrees.

**Figure 5:**
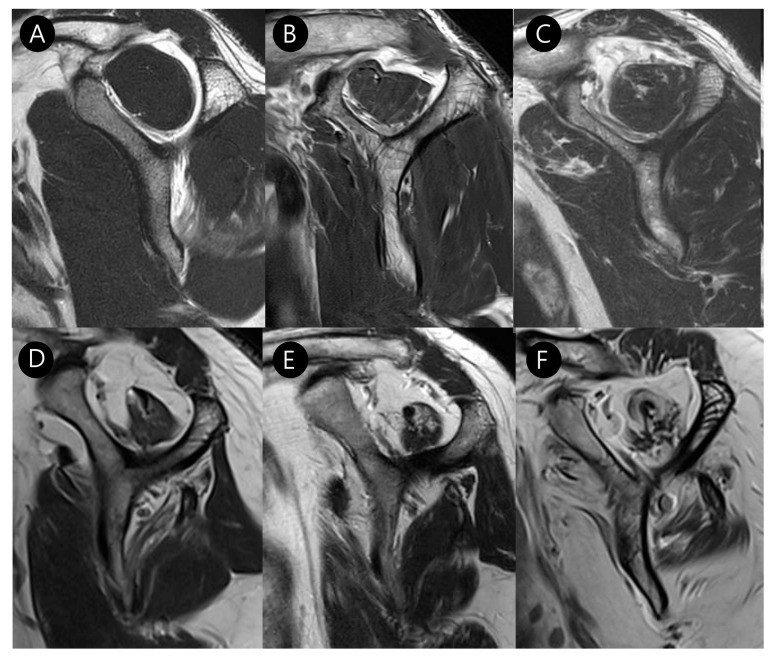
Evaluation of subscapularis muscle fatty infiltration on T2-weighted oblique sagittal images according to the modified Goutallier method [14]: (**A**) Grade 0: no fat. (**B**) Grade 1: some fatty streaks. (**C**) Grade 2: less fat than muscle in the upper half of the muscle. (**D**) Grade 3: more fat than muscle in the upper half of the muscle, with normal or fatty streaks in the lower half of the muscle. (**E**) Grade 4: more fat than muscle in the upper half of the muscle, with less fat than muscle in the lower half of the muscle. (**F**) Grade 5: more fat than muscle in the upper and lower halves of the muscle.

Fatty infiltration for the subscapularis muscle was assessed using the modified Goutallier method proposed by Yoon et al. [14]. In this system, the degree of fatty infiltration is graded separately for the upper and lower portions of the muscle (Table 2). Representative MRI examples of each modified Goutallier grade are presented in Figure 5. For statistical analysis, we categorized fatty infiltration into two categories: grade 0–1 and grade 2–3. No cases in this study showed grade 4 or higher fatty infiltration.

**Figure 6:**
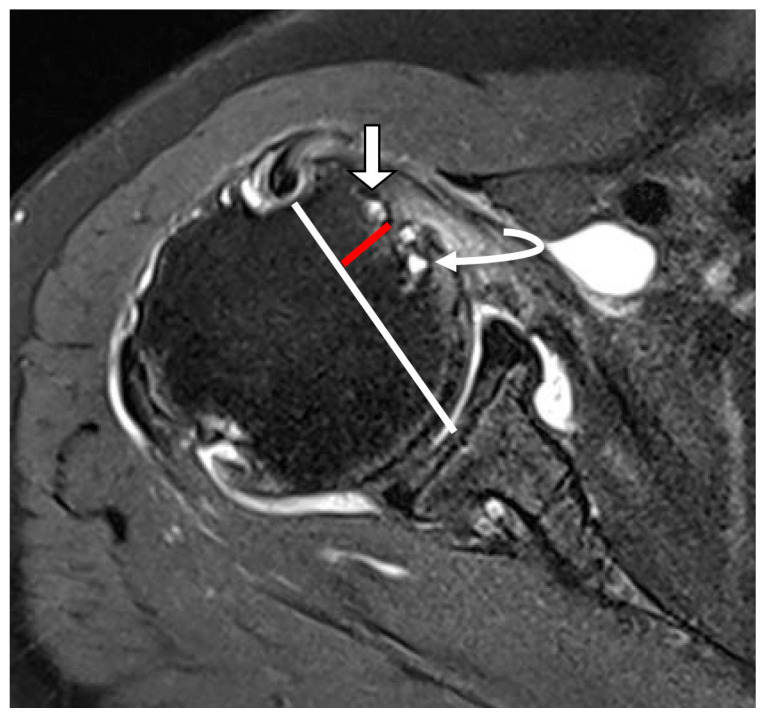
Cystic lesion in the lesser tuberosity on T2-weighted oblique axial fat-suppressed image. A white line extends from the bicipital groove to the midpoint of the glenoid fossa at the level of the lesser tuberosity. A red line indicates the border of a bony protuberance called the lesser tuberosity. Cysts located within the bony protuberance were defined as cysts within the lesser tuberosity (straight arrow). Cysts adjacent to the bony protuberance were defined as cysts adjacent to the lesser tuberosity (curved arrow).

Cysts around the lesser tuberosity were assessed on oblique axial T2-weighted fat-suppressed images, because previous studies have shown that cysts within the lesser tuberosity are highly specific indicators of SSC abnormalities [15,16]. A reference line was drawn from the bicipital groove to the midpoint of the glenoid fossa at the level of the lesser tuberosity. The border of the bony protuberance of the lesser tuberosity was outlined. Only cysts located within the lesser tuberosity were included in the analysis; cysts adjacent to the lesser tuberosity were excluded (Figure 6) [16].

**Figure 7:**
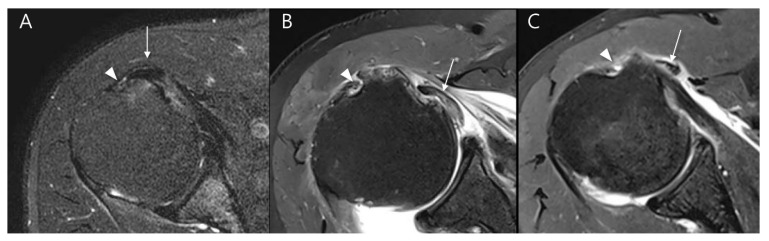
Evaluation of long head of the biceps tendon (LHBT) instability on T2-weighted oblique axial fat-suppressed images in relation to the bicipital groove: (**A**) LHBT (arrow) is partially displaced to the medial margin of the bicipital groove (arrowhead) without complete dislocation, consistent with subluxation. (**B**) LHBT (arrow) is completely displaced medially from the bicipital groove (arrowhead) and lies within the glenohumeral joint space, beneath the subscapularis tendon, representing an intra-articular dislocation. (**C**) LHBT (arrow) is completely displaced medially from the bicipital groove (arrowhead) and is located outside the joint capsule, overlying the subscapularis muscle, representing an extraarticular dislocation.

We evaluated the long head of the biceps tendon (LHBT) for various pathologies, including tendinosis, partial tears, complete tears, and instability (subluxation or dislocation) [17]. Tendinosis was defined as an increase in intratendinous signal intensity on T2-weighted images without obvious fiber discontinuity. Partial tears were characterized by focal discontinuity of tendon fibers, whereas complete tears were diagnosed when there was full thickness discontinuity or absence of the tendon in the bicipital groove [18]. Instability of the LHBT, including subluxation and dislocation (Figure 7), was assessed based on abnormal tendon positioning relative to the bicipital groove [19].

### 2.4. Statistical Analyses

All statistical analyses were performed using SPSS version 26.0 (IBM Corp., Armonk, NY, USA). To assess differences in demographic, clinical data, and MRI features between SSC tear classification groups (Yoo type I + IIA vs. Yoo type IIB), statistical comparisons were conducted using the Mann–Whitney U test for age due to non-normal distribution, and Chi-square tests for categorical variables. Interobserver agreement was analyzed by Cohen’s Kappa coefficient (κ value) for categorical variables of MRI features. Associations between MRI features and SSC tear of Yoo type IIB were investigated using multivariate logistic regression analysis to identify significant predictors. To reduce the risk of type I error from multiple comparisons, the Bonferroni correction was applied, resulting in an adjusted significance threshold of *p* < 0.00625. A *p* value below this threshold was considered statistically significant for MRI findings.

## 3. Results

### 3.1. Patient Demographics

Among Lafosse type 1 patients, the control group consisted of Yoo type I (n = 70) and Yoo type IIA (n = 41) lesions (total n = 111; median age, 63 years [range, 32–82 years]; 48 men), while the study group comprised Yoo type IIB lesions (n = 32; median age, 66 years [range, 50–85 years]; 16 men). A significant difference was observed in age between the two groups (*p* = 0.025), whereas gender distribution did not differ significantly (*p* = 0.634) (Table 3).

### 3.2. MRI Findings

Preoperative MRI findings demonstrated significant differences between the groups (Table 4). When applying Bonferroni correction for multiple comparisons (adjusted significance threshold, *p* < 0.00625), the associations of Yoo type IIB with subscapularis muscle atrophy (*p* < 0.001), subscapularis fatty infiltration of (*p* < 0.001), and LHBT pathologies including partial thickness tears (*p* < 0.001), tendinosis (*p* = 0.003), and subluxation or dislocation (*p* < 0.001) remained statistically significant. In contrast, the associations with lesser tuberosity cyst (*p* = 0.033) and LHBT full thickness tears (*p* = 0.040) did not remain significant after correction.

Interobserver agreement for Yoo and Rhee classification was substantial (κ = 0.720, *p* < 0.001). Interobserver agreement for all other MRI features was excellent, as follows (Table 4): Subscapularis muscle atrophy (κ = 1.000, *p* < 0.001) and lesser tuberosity cysts (κ = 1.000, *p* < 0.001) demonstrated perfect agreement. Subscapularis fatty infiltration (κ = 0.818, *p* < 0.001) showed almost perfect agreement. LHBT pathologies demonstrated high reliability, including full thickness tear (κ = 1.000, *p* < 0.001; excellent agreement), partial thickness tear (κ = 0.981, *p* < 0.001; almost perfect agreement), tendinosis (κ = 0.899, *p* < 0.001; almost perfect agreement), and subluxation/dislocation (κ = 0.853, *p* < 0.001; almost perfect agreement).

### 3.3. Predictive MRI Factors for Yoo Type IIB

Univariate logistic regression analysis revealed that several MRI findings were significantly associated with Yoo type IIB lesions. Older age (odds ratio [OR], 1.060; 95% confidence interval [CI], 1.010–1.113; *p* = 0.019), subscapularis muscle atrophy (OR, 57.619; 95% CI, 7.058–470.362; *p* < 0.001), subscapularis fatty infiltration (OR, 15.260; 95% CI, 2.989–77.921; *p* = 0.001), presence of a lesser tuberosity cyst (OR, 2.616; 95% CI, 1.157–5.914; *p* = 0.021), LHBT full thickness tear (OR, 4.954; 95% CI, 1.245–19.708; *p* = 0.023), LHBT partial thickness tear (OR, 15.957; 95% CI, 3.596–70.806; *p* < 0.001), LHBT tendinosis (OR, 11.093; 95% CI, 1.443–85.291; *p* = 0.021), and LHBT subluxation (OR, 31.815; 95% CI, 10.267–98.593; *p* < 0.001) were all significant predictors.

**Figure 8:**
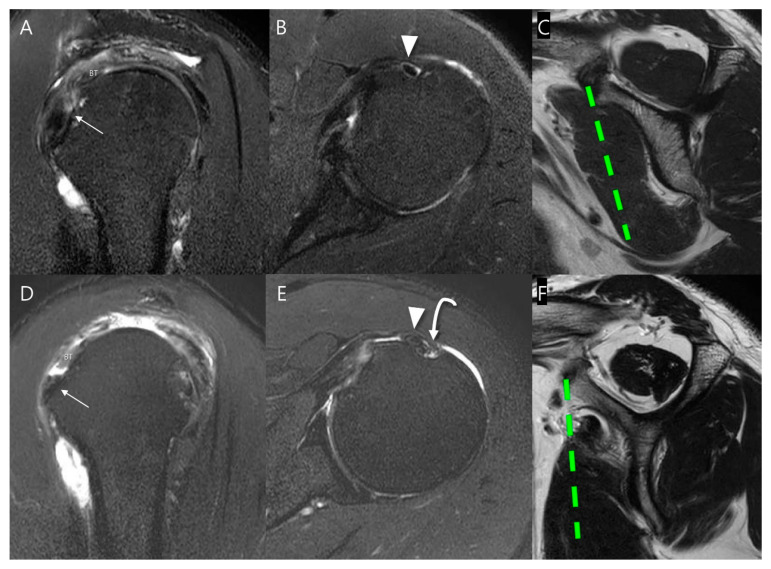
Representative MR images of Yoo type IIA (**A**–**C**) and IIB (**D**–**F**) subscapularis tendon (SSC) tears: (**A**) T2-weighted oblique sagittal fat-suppressed image in a patient with a Yoo type IIA lesion demonstrates a partial thickness tear of the SSC with ≤50% detachment from the first facet (arrow). (**B**) T2-weighted oblique axial fat-suppressed image shows an intact long head of the biceps tendon (LHBT, arrowhead) in the bicipital groove. (**C**) The T2-weighted oblique sagittal image shows no muscle atrophy. (**D**) T2-weighted oblique sagittal fat-suppressed image in a patient with a Yoo type IIB lesion demonstrates a partial thickness tear of the SSC > 50% detachment from the first facet (arrow). (**E**) T2-weighted oblique axial fat-suppressed image shows subluxation of LHBT (arrowhead) from the bicipital groove (curved arrow). (**F**) T2-weighted oblique sagittal image shows moderate muscle atrophy, especially in the upper half. BT, biceps tendon.

On multivariate logistic regression analysis, however, only subscapularis muscle atrophy (OR, 33.830; 95% CI, 2.505–456.914; *p* = 0.008) and LHBT subluxation (OR, 22.836; 95% CI, 3.993–130.605; *p* < 0.001) remained as independent predictors of Yoo type IIB lesions. The multicollinearity of the independent variables in the logistic regression model was assessed using the variance inflation factor (VIF). All predictor variables showed low VIF (<2), indicating a general absence of multicollinearity, except for LHBT subluxation/dislocation, which exhibited a VIF of 6.75. The detailed results are summarized in Table 5. Representative MR images from the control and study groups are presented in Figure 8.

### 3.4. Flowchart of MRI Predictors for Yoo Subclassification

**Figure 9:**
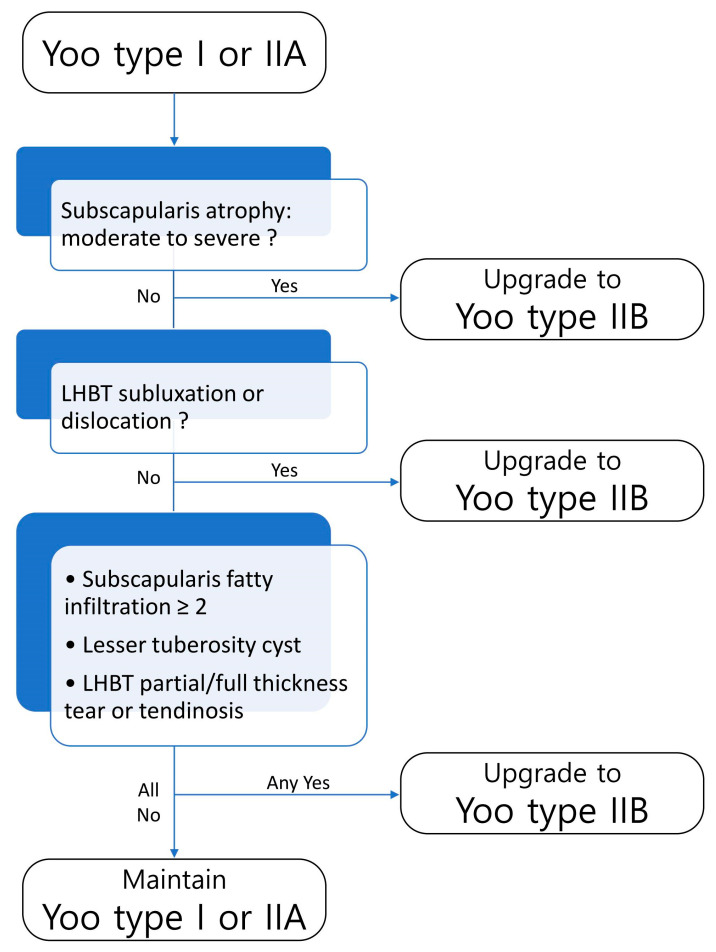
Decision tree for Yoo and Rhee subclassification. Lesions are upgraded to Yoo type IIB when any of the following are present: moderate to severe subscapularis atrophy, LHBT subluxation/dislocation, subscapularis fatty infiltration ≥ 2, lesser tuberosity cyst, or LHBT partial/full thickness tear or tendinosis. If none of these findings are observed, the lesion remains as Yoo type I or IIA.

We developed a decision tree to assist in upgrading Yoo type I or IIA to type IIB based on MRI findings (Figure 9). When moderate to severe subscapularis muscle atrophy is present in lesions suspected as Yoo type I or IIA, the lesion could be upgraded to Yoo type IIB. The presence of LHBT subluxation or dislocation warrants an upgrade to type IIB. In the absence of these findings, additional features, including subscapularis fatty infiltration grade ≥ 2, lesser tuberosity cyst, or LHBT partial/full thickness tear or tendinosis, may also indicate an upgrade to Yoo type IIB. If none of these findings are met, the lesion remains as Yoo type I or IIA.

## 4. Discussion

In this study, we investigated the MRI characteristics associated with partial thickness SSC tears. Our study divided partial thickness SSC tears into two groups: Yoo type I + IIA vs. Yoo type IIB. Our findings demonstrated that patients with Yoo type IIB lesions were significantly older than those with Yoo type I + IIA lesions, although gender distribution did not differ between groups. MRI evaluation revealed a higher prevalence of subscapularis muscle atrophy, fatty infiltration, and lesser tuberosity cysts in the Yoo type IIB group, along with more frequent LHBT pathologies, including partial and full thickness tears, tendinosis, and instability. Notably, multivariate logistic analysis identified subscapularis muscle atrophy and LHBT subluxation as independent MRI predictors of Yoo type IIB lesions, highlighting their potential role in the pathogenesis and progression of more advanced SSC tears.

While the Lafosse classification has been the standard for describing SSC tears, it is primarily based on arthroscopic findings and lacks the detailed subclassification needed to guide surgical decisions for partial tears [8,20]. This study addressed this gap by evaluating the Yoo and Rhee classification, which subdivides Lafosse type 1 lesions based on the facet anatomy of the SSC footprint, and identifying MRI predictors for lesions that require surgical repair [7,21,22]. This study successfully identified key MRI findings that are significantly associated with Yoo type IIB tears, a new surgical indicator defined as >50% detachment of the first facet. Our findings demonstrated that patients with Yoo type IIB tears had a higher prevalence of subscapularis muscle atrophy, fatty infiltration, lesser tuberosity cysts, and LHBT pathologies compared to those with Yoo type I + IIA tears. This aligns with previous research that highlights the clinical and imaging utility of the Yoo and Rhee classification.

By distinguishing partial thickness SSC tears according to the Yoo and Rhee classification, our study highlights significant structural differences that may influence clinical management. Our multivariate logistic regression analysis confirmed that the higher prevalence of subscapularis muscle atrophy and LHBT subluxation in Yoo type IIB lesions than in the control group (Yoo type I + IIA) may serve as surgical indications for partial thickness SSC tears. These findings are consistent with prior studies reporting that muscle quality and LHBT status are critical determinants of repair outcomes in SSC tears [23,24,25,26]. Subscapularis muscle atrophy, characterized by fatty infiltration and muscle degeneration, has been associated with poor functional outcomes and increased risk of tendon retear following rotator cuff repair [27,28]. Similarly, LHBT subluxation, often observed in conjunction with SSC tears, can lead to persistent anterior shoulder pain and may necessitate surgical intervention [29,30]. The presence of these MRI findings in Yoo type IIB lesions aligns with criteria for surgical management of SSC tears, emphasizing a valuable framework for radiologists and orthopedic surgeons to improve preoperative diagnostic accuracy and guide surgical planning. It should be noted, however, that the recommendation of Yoo type IIB lesions as surgical indications has been largely informed by the original work of Yoo and colleagues and its subsequent application in clinical practice, rather than by an international consensus.

Despite these valuable findings, several limitations should be acknowledged. First, this study was conducted retrospectively at a single center, which may limit the generalizability of the results. Moreover, detailed information regarding patients’ occupation or participation in shoulder-involving sports or leisure activities was not collected in this retrospective study, which may affect the generalizability of the findings. In addition, the Yoo and Rhee classification was determined based only on MRI findings and was not confirmed by arthroscopic findings. Second, the sample size, particularly for Yoo type IIB lesions, was relatively small, potentially affecting the statistical power of the analyses. Post hoc power analysis indicated that the study had limited ability to detect small differences in demographic data such as age (Cohen’s d = 0.32; power ≈ 0.53) and sex distribution (Cohen’s h = 0.14; power ≈ 0.12). However, for the MRI findings, the observed effect sizes were moderate to very large (Cohen’s h range, 0.71–1.15), and post hoc power exceeded 0.8, supporting the robustness of the MRI factors despite the group size imbalance. Additionally, given the difference in median age between groups, age may act as a confounder affecting both muscle quality and LHBT pathologies, which should be considered when interpreting the results. Third, the strong correlation between subscapularis muscle atrophy and fatty infiltration (r = 0.758, *p* < 0.001) was carefully considered. By including both variables in multivariable logistic regression, we confirmed that each factor was independently associated with Yoo type IIB lesions (atrophy: adjusted OR = 48.34, 95% CI = 5.60–417.14; fatty infiltration: adjusted OR = 11.52, 95% CI = 1.90–69.99). Fourth, the VIF for the LHBT subluxation/dislocation was 6.75, which suggests a degree of multicollinearity, possibly stemming from its co-occurrence with other LHBT pathologies included in the model. Although this value exceeds the common VIF threshold of 5, it remains within the range (5 to 10) often considered tolerable. Furthermore, we selected to retain this factor in the predictive model because LHBT instability is regarded as one of the most representative findings among the LHBT conditions. Fifth, perfect interobserver agreement (κ = 1.000) was observed for subscapularis muscle atrophy, lesser tuberosity cysts, and full thickness tears of LHBT. However, these values were obtained after dichotomization of the MRI findings, and the choice of threshold (e.g., non-mild vs. moderate–severe atrophy) may have contributed to the perfect agreement. Sixth, reliable bootstrapped CIs for the odds ratios could not be obtained due to sparse data distributions and frequent convergence failures in the logistic regression models, particularly for LHBT-related variables, which may have affected the stability of the effect size estimates. Lastly, the study did not evaluate long-term functional outcomes after surgical repair, which would be important to confirm the clinical significance of the identified MRI predictors. Future prospective multicenter studies with larger cohorts and inclusion of post-operative functional outcomes are warranted to validate the clinical utility of these MRI features in guiding surgical intervention.

## 5. Conclusions

Our study provides a valuable framework for improving the preoperative diagnosis of partial thickness SSC tears and important insights into the MRI features associated with Yoo type IIB lesions as surgical indicators. The identification of subscapularis muscle atrophy and LHBT subluxation as independent predictors offers a practical framework for preoperative assessment, enabling radiologists and orthopedic surgeons to more accurately stratify patients who may benefit from surgical repair. These findings emphasize the value of detailed MRI evaluation in guiding clinical decision-making and optimizing patient outcomes.

## Figures and Tables

**Table 1:** Yoo and Rhee classification of subscapularis tendon (SSC) tears.

Type I	Fraying or longitudinal split of the leading edge tendon
Type II	Partial detachment in the first facet (Lafosse type 1)
IIA	≤50% detachment of SSC in the first facet
IIB	>50% detachment of SSC in the first facet without complete disruption of the lateral band
Type III	Complete first facet detachment with disruption of the lateral band (full thickness superior 1/3 of SSC tear, Lafosse type 2)
Type IV	First and second facets exposed with medial retraction of the subscapularis (equal to upper 2/3 of SSC tear, Lafosse type 3)
Type V	Complete SSC tear involving the muscular portion

**Table 2:** Modified Goutallier classification for fatty infiltration of the subscapularis muscle.

Modified Goutallier Classification [14]	
Grade 0	No fat
Grade 1	Some fatty streaks
Grade 2	Less fat than muscle in the upper half
Grade 3	More fat than muscle in the upper half, with no fat or fatty streaks in the lower half
Grade 4	More fat than muscle in the upper half, with less fat than muscle in the lower half
Grade 5	More fat than muscle in both upper and lower halves

**Table 3:** Patient characteristics between the two groups.

	Control Group (Yoo Type I + IIA, n = 111)	Study Group (Yoo Type IIB, n = 32)	*p* Value
Age (years), median [range]	63 [32–82]	66 [50–85]	0.025
Sex			0.634
Male (%)	48 (43.2)	16 (50.0)	
Female (%)	63 (56.8)	16 (50.0)	

**Table 4:** MRI image analysis between the two groups and interobserver agreements.

	Control Group (Yoo Type I + IIA, n = 111)	Study Group (Yoo Type IIB, n = 32)	*p* Value	Interobserver Agreement (*p* Value)
Subscapularis muscle atrophy			<0.001	κ = 1.000 (*p* < 0.001)
None to mild degrees (%)	110 (99.1)	21 (65.6)		
Moderate to severe degrees (%)	1 (0.9)	11 (34.4)		
Subscapularis muscle fatty infiltration			<0.001	κ = 0.818 (*p* < 0.001)
None to some fatty streaks (grade 0 and 1, %)	109 (98.2)	25 (78.1)		
Less or more than 50% of fat than muscle (grade 2 and 3, %)	2 (1.8)	7 (21.9)		
Lesser tuberosity cyst (%)	28 (25.2)	15 (46.9)	0.033	κ = 1.000 (*p* < 0.001)
Long head biceps tendon pathologies				
Full thickness tear (%)	4 (3.6)	5 (15.6)	0.040	κ = 1.000 (*p* < 0.001)
Partial thickness tear (%)	47 (43.9)	25 (92.6)	<0.001	κ = 0.981 (*p* < 0.001)
Tendinosis (%)	76 (71.0)	27 (100.0)	0.003	κ = 0.899 (*p* < 0.001)
Subluxation or dislocation (%)	13 (12.1%)	22 (81.5%)	<0.001	κ = 0.853 (*p* < 0.001)

**Table 5:** Predictive MRI factors predicting Yoo type IIB.

	Univariate			Multivariate		
	Odds Ratio	95% CI	*p* Value	Odds Ratio	95% CI	*p* Value
Age	1.060	1.010–1.113	0.019	-	-	-
Sex	0.762	0.346–1.676	0.499	-	-	-
Subscapularis muscle atrophy	57.619	7.058–470.362	<0.001	33.830	2.505–456.914	0.008
Subscapularis muscle fatty infiltration	15.260	2.989–77.921	0.001	-	-	-
Lesser tuberosity cyst	2.616	1.157–5.914	0.021	-	-	-
LHBT full thickness tear	4.954	1.245–19.708	0.023	-	-	-
LHBT partial thickness tear	15.957	3.596–70.806	<0.001	-	-	-
LHBT tendinosis	11.093	1.443–85.291	0.021	-	-	-
LHBT subluxation	31.815	10.267–98.593	<0.001	22.836	3.993–130.605	<0.001

CI: confidence interval.

## Data Availability

The dataset is available upon request from the authors.
